# Hepatocellular- cholangiocarcinoma collision tumor: a case report

**DOI:** 10.3389/fonc.2026.1860896

**Published:** 2026-09-25

**Authors:** Hongzhi Cao, Lin Long, Canbin Xie, Yu Liu, Jianqi Zou, Lin Sun

**Affiliations:** The Interventional Vascular Surgery, Hunan Provincial People’s Hospital (Hunan Normal University First Hospital), Changsha, Hunan, China

**Keywords:** bevacizumab, cisplatin, collision tumor, gemcitabine, hepatocellular carcinoma (HCC), intrahepatic cholangiocarcinoma (ICC), sintilimab, transcatheter arterial chemoembolization (TACE)

## Abstract

Hepatocellular-cholangiocarcinoma collision tumor is an extremely rare primary liver malignancy, defined as histologically distinct hepatocellular carcinoma (HCC) and intrahepatic cholangiocarcinoma (ICC) growing adjacently with clear demarcation, which is easily misdiagnosed due to overlapping features with combined hepatocellular-cholangiocarcinoma and metastatic liver cancer, and lacks standardized diagnostic and treatment evidence. We report a 54-year-old male with 15-year chronic hepatitis B infection admitted in June 2024 with fatigue, poor appetite and abdominal distension. Preoperative CT initially suggested multifocal metastatic HCC, while Gd-EOB-DTPA MRI indicated dual primary tumors, and multi-target biopsy confirmed HCC-ICC collision tumor (S8 segment grade III HCC, S6 segment moderately differentiated ICC, no mixed components). After multidisciplinary team (MDT: Oncology Department, Interventional Vascular Surgery Department, Hepatobiliary Surgery Department, Radiology Department, Internal Medicine for Liver Diseases Department, Infectious Diseases Department) discussion, the patient was ineligible for surgery due to lymph node metastases, received transarterial chemoembolization for the HCC lesion, followed by sequential PD-1 inhibitor(Sintilimab) plus bevacizumab and 8 cycles of gemcitabine plus cisplatin. As of the last follow-up on April 20, 2026, the patient had an overall survival of over 18 months with partial response lesions and no severe adverse events. This case indicates that HCC-ICC collision tumor should be considered in the differential diagnosis of multifocal liver malignancies, multi-target biopsy is critical for accurate diagnosis, and individualized combination therapy targeting both components can achieve favorable survival outcomes, adding to the clinical evidence for this rare disease.

## Introduction

Hepatocellular-cholangiocarcinoma collision tumor and combined hepatocellular-cholangiocarcinoma (cHCC-CCA) represent two distinct categories of liver malignancies. Both exhibit differentiation characteristics of hepatocellular carcinoma (HCC) and cholangiocarcinoma; however, they possess fundamentally different pathological natures. These differences contribute to a high propensity for misdiagnosis in clinical practice and are evident across multiple dimensions.

Recent studies confirm that cHCC-CCA is a mixed tumor of monoclonal origin, characterized by interwoven components (1–3). This tumor type has been classified as an independent disease entity in the 2019 WHO Classification of Digestive System Tumors (4).In contrast, collision tumors are dual-origin neoplasms formed by two independent primary tumors that grow adjacent to one another in the liver. These tumors exhibit clear boundaries and lack histological transition zones or mixed components, thus excluding them from the cHCC-CCA category (5). Furthermore, both tumor types are susceptible to misinterpretation in preoperative imaging. The diagnosis of cHCC-CCA necessitates confirmation of dual differentiation features within the same tumor through hematoxylin-eosin (HE) staining and immunohistochemistry (6),whereas collision tumors require pathological evidence of independent growth boundaries and the absence of component mixing. Currently, there is no standardized clinical consensus for the differential diagnosis of these two tumor types, and accurate diagnosis depends on comprehensive multi-target pathological sampling and analysis (7). Additionally, the prognosis for cHCC-CCA lies between that of HCC and ICC, with an increased risk of lymph node metastasis (8).As dual primary tumors, treatment strategies for collision tumors must be developed to target each tumor component separately, making precise differentiation between the two critical for surgical planning, systemic treatment selection, and prognostic evaluation. Against this background, we report a rare case of pathologically confirmed HCC-ICC collision tumor, aiming to summarize the key points of differential diagnosis between collision tumor and cHCC-CCA, and explore the efficacy of individualized combination treatment strategies targeting both tumor components, so as to provide reference for clinical diagnosis and treatment of this rare disease.

## Case presentation

A 54-year-old male patient was admitted on June 12, 2024, presenting with “fatigue, poor appetite, and intermittent abdominal distension for 1 week.” He has a 15-year history of chronic hepatitis B virus (HBV) infection. Physical examination revealed features consistent with chronic liver disease, mild tenderness in the upper abdomen, and the absence of rebound tenderness or abdominal muscle rigidity. Routine blood tests conducted upon admission indicated pancytopenia, and liver function was classified as Child-Pugh grade A. Tumor marker tests revealed elevated levels: alpha-fetoprotein (AFP) at 97.52 ng/ml (reference range 0–7 mAU/ml), protein induced by vitamin K absence or antagonist-II (PIVKA-II) at 555.72 mAU/ml (reference range ≤40 mAU/ml), carbohydrate antigen 19-9 (CA19-9) at 2 U/ml (reference range 0–37 U/ml), and carcinoembryonic antigen (CEA) at 44.32 ng/ml (reference range ≤3.88 ng/ml),the patient’s liver function was Child-Pugh grade A, with total bilirubin 18.2 μmol/L, albumin 41.3 g/L, alanine aminotransferase 42 U/L, aspartate aminotransferase 38 U/L, prothrombin time 11.2 s, and no history of ascites or hepatic encephalopathy. Contrast-enhanced CT scans demonstrated multiple slightly hypodense space-occupying lesions in the right liver, highly suggestive of HCC with metastasis. The imaging also indicated involvement of the right portal vein branch and right hepatic vein, as well as multiple enlarged retroperitoneal lymph nodes, liver cirrhosis, splenomegaly, and ascites. Hepatobiliary-specific Gd-EOB-DTPA (Primovist) enhanced MRI revealed a nodule in liver segment S8, which was classified as HCC with involvement of the right portal vein branch and the right hepatic vein. Additionally, a nodule in liver segment S6 was identified as a potential ICC, along with multiple retroperitoneal lymph nodes suggestive of metastases, liver cirrhosis was also indicated (**Figure 1**). Pathological biopsy results (**Figure 2**) indicated that: ① the S6 liver nodule was diagnosed as moderately differentiated cholangiocarcinoma, with IHC showing CK(+) and CK19(+), while Hepatocyte(-), CK7 (partially +), Arg-1 (-), and Ki67 (+, 20%); ② the S8 liver nodule was identified as grade 3 HCC, with IHC results showing CK7 (partially +), CK19 (-), Hepatocyte (-), Arg-1 (partially weakly +), and Ki67 (+, 10%). The surgical team determined that the patient was not a candidate for surgery. The patient’s HCC component accounted for most of the total tumor volume, was of higher histological grade, which was assessed as the dominant subtype driving tumor progression. Therefore, after multidisciplinary team (MDT) discussion, we chose the first-line standard regimen for advanced HCC (PD-1 inhibitor combined with bevacizumab) as the systemic treatment, combined with TACE to control the intrahepatic dominant lesion. The oncology team then initiated therapy with a PD-1 inhibitor combined with bevacizumab, followed by eight cycles of gemcitabine plus cisplatin as anti-tumor treatment. The patient is currently under follow-up, with stable control of the lesions (**Tables 1**–**3**). During the treatment, the patient developed grade 1 hypertension and grade 2 thrombocytopenia, which were controlled by symptomatic treatment without dose reduction or treatment interruption. No grade 3 or above serious adverse events occurred.

**Figure 1:**
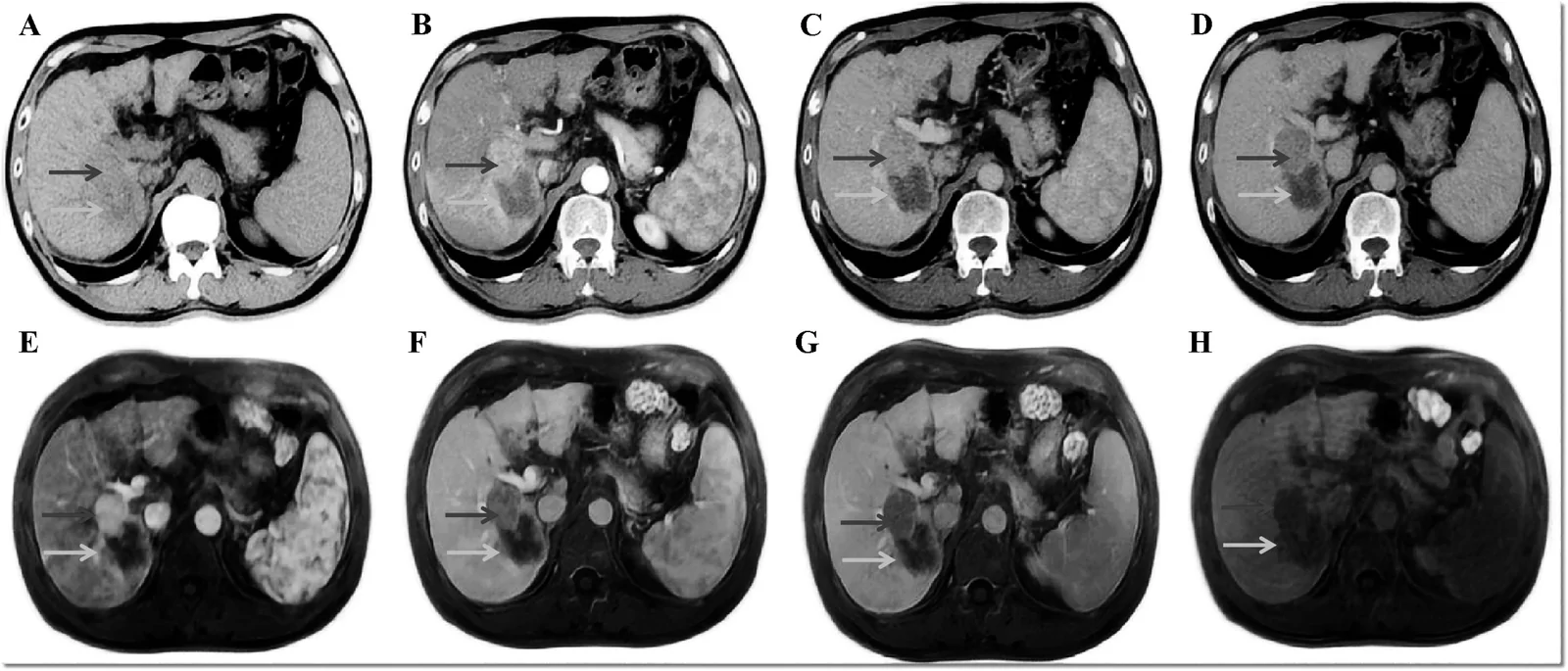
Preoperative axial liver images. **(A–D)** Non-contrast and contrast-enhanced CT images in different phases; **(E–H)** Contrast-enhanced MRI images in different phases, including gadoxetic acid (Primovist)-enhanced images. A mass‑like lesion with heterogeneous density is seen in segments 6/8, showing regional enhancement across all contrast‑enhanced phases, with clear demarcation between the different enhancing components and no evidence of fusion or transitional zones.

**Figure 2:**
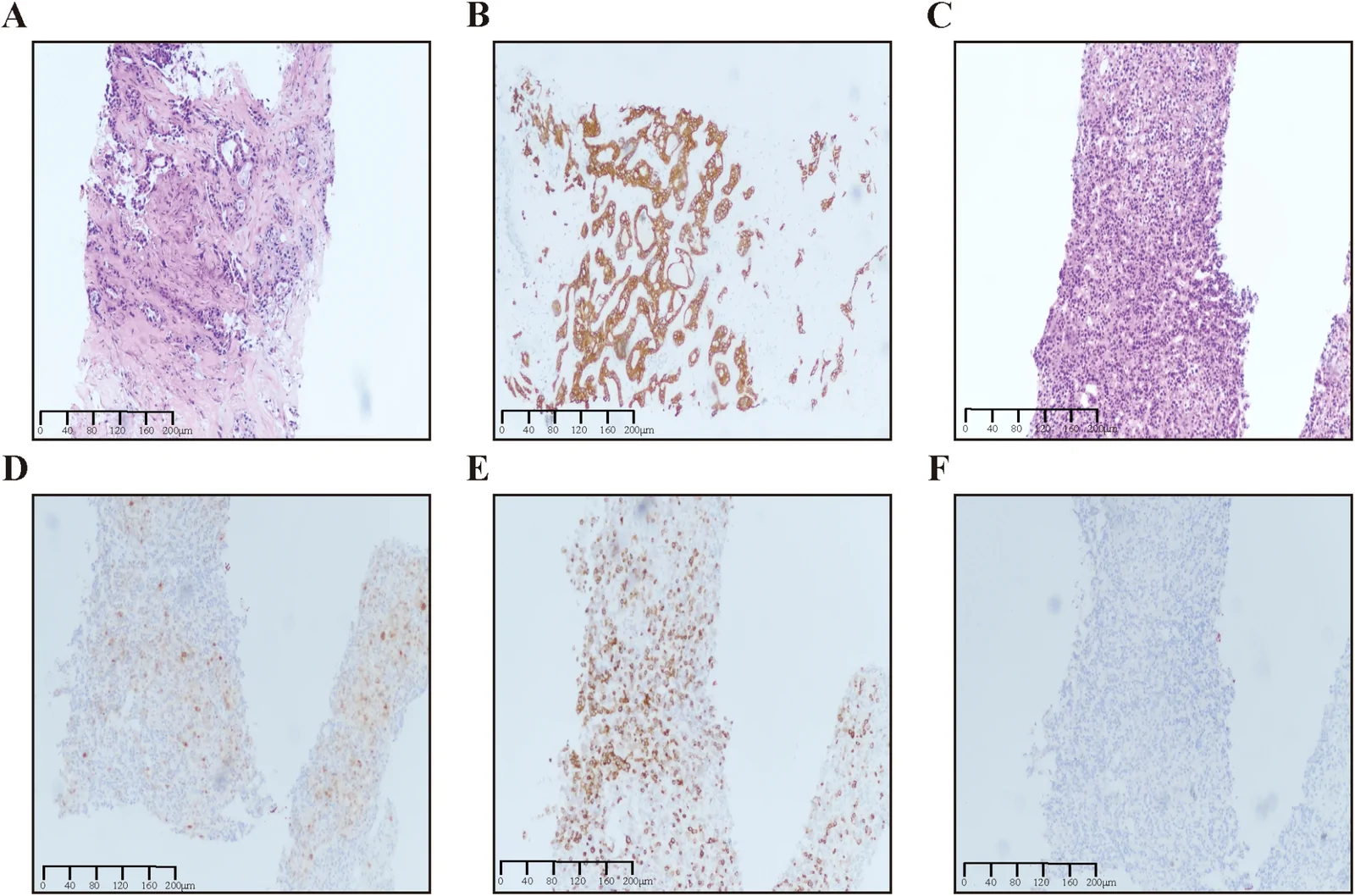
**(A,B)** background ICC: **(A)** HE,Magnication,x10; **(B)** CK19(+),Magnication,x10; **(C–F)** background HCC: **(C)** HE,Magnication,x10; **(D)** Arg(partially +),Magnication,x10; **(E)** CK7(partially +),Magnication,x10; **(F)** CK19(-),Magnication,x10.

**Table 1:** Treatment regimen and dosing schedule.

Time	Sintilimab	Bevacizumab	Gemcitabine	Cisplatin
01/09/2025	TACE			
01/24/2025	200	870		
02/14/2025	200	870		
03/06/2025	200	870		
03/31/2025	200	870		
04/27/2025			1700	42
05/18/2025			1360	33
06/10/2025			1360	33
06/30/2025			1200	42
08/15/2025	200	870		
09/05/2025			1200	42
09/28/2025	200	870		
10/30/2025			1200	42
11/20/2025	200	870		
12/29/2025	200	870		
01/20/2026			1200	42
02/12/2026	200	870		
03/12/2026	200	700		
04/14/2026	200	700		

Unit: mg.

**Table 2:** Efficacy evaluation.

Time	ICC(S6)	HCC(S8)	Extrahepatic metastasis	RECIST1.1
08/30/2024	47	37	25	–
01/17/2025	68	37	21	SD
04/19/2025	48	26	11	SD
06/09/2025	43	20	10	PR
08/13/2025	35	20	9	PR
12/09/2025	35	15	5	PR

Unit: mm.

ICC, Intrahepatic Cholangiocarcinoma; HCC, Hepatocellular Carcinoma; RECIST1.1, Response Evaluation Criteria in Solid Tumors, version 1.1; PR, Partial Response; SD, Stable Disease.

**Table 3:** Tumor markers.

Time	Abnormal thrombin(≤40~)	AFP(ng/ml)(0~8.78)
09/24/2024	7048.52	58.63
01/08/2025	3733.07	10.69
01/15/2025	7119.74	11.35
01/23/2025	8088.11	12.42
02/11/2025	790.74	13.12
03/04/2025	1402.98	14.33
03/31/2025	682.66	8.85
04/27/2025	244.82	10.43
07/25/2025	464.12	7.79
05/15/2025	434	7.51
06/09/2025	381.38	8.35
06/28/2025	303.11	8.42
08/30/2025	304.62	7.73
11/20/2025	173.89	7.73
12/29/2025	270.27	7.85
01/20/2026	555.72	6.09
02/12/2026	445.44	7.46
03/12/2026	440.82	6.56
04/14/2026	839.4	6.42

## Discussion

HCC-ICC collision tumor is extremely rare entity. To the best of our knowledge, only a very small number of cases meeting the strict definition have been reported in the literature to date (9, 10)(**Table 4**). Notably that there is certain heterogeneity in previously reported cases, some of which included mixed tumor components or multi-lineage differentiation components, which do not fully adhere to the standard diagnostic criteria of pure collision tumor. This study contributes valuable clinical data on this uncommon condition, enhancing awareness of the differential diagnosis for multifocal liver malignancies.

**Table 4:** Summary of key references on collision tumors.

Age	Gender	Underlying liver disease	Tumor components	Clear demarcation	Treatment	Clinical outcome	Author & year of publication
12	Male	Jaundice	HCC and cholangiocarcinoma	/	liver transplant	Currently well	Krishnan, Sathish K,2022
69	Male	hepatitis C	HCC and ICC	Yes	Sytemic therapy &laparoscopic Middle hepatectomy with cholecystectomy		He YC,2025

The patient in this case had a prolonged history of chronic HBV infection, a primary risk factor for liver carcinogenesis. Current research indicates that HBV-induced HCC operates through dual “direct-indirect” mechanisms: viral integration into the host genome can disrupt tumor suppressor genes, activate proto-oncogenes, induce chromosomal aberrations, genomic instability, and insertional mutagenesis, potentially leading to multifocal HCC clonal origin even in cases of occult infection without viral replication (11), HBV proteins, including HBx, directly disrupt host genomic stability and signaling pathways, thereby fostering a pro-tumor microenvironment through indirect mechanisms such as chronic inflammation, immune exhaustion, and metabolic disorder (12). In the context of ICC, current evidence indicates that HBV contributes to ICC carcinogenesis via multiple pathways, including virus integration-induced host genomic abnormalities, high viral load-driven chronic injury, and hepatocyte transdifferentiation (13, 14), However, the specific molecular pathways and immune regulatory mechanisms by which HBV targets the biliary epithelium remain to be elucidated. In this case, the collision tumor was pathologically confirmed as a dual-origin neoplasm, suggesting that the patient experienced concurrent carcinogenic mechanisms for both HCC and ICC.

The clinical features of this patient aligned with the corresponding imaging and pathological findings. The S6 segment ICC exhibited ring enhancement on CT and persistent enhancement during the portal venous phase, which corresponded to the pathological diagnosis of moderately differentiated ICC. IHC analysis revealed CK19 positivity, Hepatocyte negativity, and Arg-1 negativity. In contrast, the S8 segment HCC demonstrated significant arterial phase enhancement and rapid washout in the venous phase on CT, consistent with the pathological diagnosis of grade 3 HCC, characterized by CK19 negativity and partial weak positivity for Arg-1. The initial contrast-enhanced CT mistakenly diagnosed the condition as HCC with metastasis. However, subsequent Gd-EOB-DTPA enhanced MRI indicated the presence of dual-origin tumors of HCC and ICC. This finding underscores the inadequacy of a single imaging modality in differentiating multifocal lesions as either single tumor metastases or dual primary collision tumors. Gd-EOB-DTPA enhanced MRI can aid in determining the origin of lesions, while multi-target puncture sampling can prevent the missed diagnosis of distinct pathological components in suspected multifocal lesions.

In the pathological diagnosis section, the immunohistochemical (IHC) basis for diagnosing the S8 nodule as hepatocellular carcinoma (HCC) is not sufficiently typical and requires clear clarification.1.Morphological evidence: As confirmed by our attending pathologists, the S8 nodule shows a typical trabecular growth pattern of HCC under hematoxylin-eosin (HE) staining. The tumor cells are medium to large in size, with an elevated nuclear-to-cytoplasmic ratio and marked nuclear atypia. No definitive bile duct-like glandular structures, goblet cells, or mucin secretion were observed, making the morphological features more consistent with HCC rather than ICC (15, 16). Two independent senior pathologists reviewed all pathological slides and reached a unanimous diagnostic conclusion.2.Interpretation of immunohistochemical results: In well-differentiated HCC, Hepatocyte (Hep Par-1) and Arginase-1 (Arg-1) are usually diffusely and strongly positive. However, it is not uncommon for one or both markers to be completely absent or only retain focal expression in poorly differentiated HCC (17). In this case, the complete loss of Hepatocyte expression and only focal weak positivity of Arg-1 are consistent with the characteristics of high-grade/poorly differentiated HCC.3.Explanation of partial CK7 positivity: Although most typical HCC cases do not express CK7, approximately 20%-30% of HCC cases may show focal or diffuse CK7 expression, especially in peribiliary regions or areas with stem cell phenotype, which does not exclude the diagnosis of HCC (15, 16).While classical markers, such as HepPar1, Arg-1, and GPC3, remain foundational for distinguishing HCC from benign lesions and metastatic tumors, their limitations in specificity and sensitivity underscore the need for combinatorial approaches. For instance, Arg-1 demonstrates superior sensitivity in poorly differentiated HCC compared to HepPar1, but its occasional reactivity in cholangiocarcinoma necessitates integration with GPC3 or pCEA (canalicular pattern specificity) (18).4.Notably, CK19 (a key biliary/stem cell marker) was negative in the S8 nodule, which is of critical value for excluding ICC or combined hepatocellular-cholangiocarcinoma (cHCC-ICC) (15). In addition, according to the Guidelines for the Diagnosis and Treatment of Primary Liver Cancer issued by the Chinese Society of Clinical Oncology, the patient has a long history of chronic hepatitis B infection, and magnetic resonance imaging (MRI) and contrast-enhanced computed tomography (CT) showed typical “fast-in and fast-out” enhancement pattern of the S8 nodule, which meets the clinical diagnostic criteria for HCC (19).

This patient was initially diagnosed with multiple lymph node metastases, resulting in a prognosis significantly worse than that of patients with localized disease, as indicated by traditional staging. The median overall survival (OS) for patients with advanced ICC is approximately 8 to 12 months, while the median OS for patients with advanced HCC accompanied by lymph node metastasis is approximately 10 to 15 months. In contrast, the survival time for patients with mixed liver cancer is typically shorter than that of the aforementioned categories (20, 21). In this case, the HCC component was managed with TACE and regular administration of a PD-1 inhibitor combined with bevacizumab, whereas the ICC component was treated with a regimen of gemcitabine and cisplatin chemotherapy. To date, the patient remains under follow-up, with an OS exceeding 18 months(last follow-up time:April 20,2026). This treatment regimen addresses the proliferative characteristics of cholangiocarcinoma through chemotherapy, which synergizes with immunotherapy and anti-angiogenic therapy, resulting in favorable efficacy.

In conclusion, hepatocellular-cholangiocarcinoma collision tumor represents an extremely rare dual primary malignant liver tumor. Based on the patient’s imaging findings (**Figure 1**) The tumor exhibits two distinct enhancement patterns coexisting without clear demarcation between adjacent regions. Subsequent pathological examination of the biopsy sample revealed that different tumor areas demonstrated distinct components, meeting the diagnostic criteria for a collision tumor. In contrast, synchronous dual primary hepatocarcinomas typically refer to two completely independent tumors located in separate liver lobes separated by normal hepatic parenchyma; the patient’s imaging data do not meet these diagnostic criteria. Therefore, we conclude this case represents a collision tumor. Although imaging features allow for preliminary differentiation from synchronous dual primary tumors, definitive diagnosis requires further evaluation. Hepatocellular-cholangiocarcinoma collision tumor’s clinical and imaging characteristics closely resemble those of cHCC-CCA and metastatic liver malignancies, resulting in elevated rates of missed and misdiagnoses. Accurate diagnosis necessitates multi-target pathological sampling and IHC analysis to confirm the presence of two distinct tumor components. Multiple targeted biopsies are recommended when imaging shows multifocal liver lesions with inconsistent imaging features (e.g., different enhancement patterns, disparate growth rates) or when serum levels of both HCC and CCA tumor markers (AFP and CA19-9) are simultaneously elevated, to avoid misdiagnosis of collision tumors as single-pathology tumors. Biopsy targets should cover all radiologically distinct lesion subtypes, and at least 2 samples should be collected from each subtype to reduce sampling bias. Consequently, collision tumors should be considered in the preoperative differential diagnosis of multifocal liver malignancies in patients with a background of chronic liver disease, in order to prevent misdiagnosis as advanced metastatic tumors and to preserve the opportunity for radical surgical intervention. Treatment regimens for confirmed cases must address the biological characteristics of both HCC and ICC, and recurrence markers for both tumor types should be monitored concurrently during post-treatment follow-up. Currently, the available case data for this condition is limited, necessitating the accumulation of larger sample sizes to elucidate optimal treatment strategies, prognostic risk factors, and long-term survival outcomes.

## Data Availability

The original contributions presented in the study are included in the article/supplementary material. Further inquiries can be directed to the corresponding author.
